# Docking of *CDK1* with antibiotic drugs revealed novel therapeutic value in breast ductal cancer *in situ*

**DOI:** 10.18632/oncotarget.18779

**Published:** 2017-06-28

**Authors:** Zhong-Hai Ding, Jia Qi, An-Quan Shang, Yu-Jie Zhang, Jun Wei, Li-Qing Hu, Wei-Wei Wang, Man Yang

**Affiliations:** ^1^ Department of Senior Cadres’ Healthcare, Jinling Hospital, Medical School of Nanjing University, Nanjing 210002, Jiangsu, China; ^2^ Department of Dermatology, Nanjing Medical University Affiliated Wuxi Second Hospital, Wuxi 214002, Jiangsu, China; ^3^ Department of Laboratory Medicine, Tongji Hospital of Tongji University, Shanghai 200092, Shanghai, China; ^4^ The Sixth People’s Hospital of Yancheng City, Yancheng 224005, Jiangsu, China; ^5^ Clinical Medicine School, Ningxia Medical University, Yinchuan 750004, Ningxia, China; ^6^ Department of Laboratory Medicine, The first Hospital of Ningbo City, Ningbo 315010, Zhejiang, China; ^7^ Department of Pathology, The First People’s Hospital of Yancheng City and The Sixth People’s Hospital of Yancheng City, Yancheng 224001, Jiangsu, China; ^8^ Department of Laboratory Medicine, TCM Hospital of Yancheng City Affiliated to Nanjing University of Chinese Medicine, Yancheng 224001, Jiangsu, China

**Keywords:** ductal cancer *in situ*, GEO, molecular signature, microarray, protein docking

## Abstract

The aim of our research is to identify potential genes associated with Ductal carcinoma *in situ* (DCIS) through microarrays. The microarray dataset GS54665 were downloaded from the GEO(Gene Expression Omnibus) database. Dysregulated genes were screened and their associations with DCIS was analyzed by comprehensive bioinformatics tools. A total of 649 differential expression genes were identified between normal and DCIS samples, including 224 up-regulated genes and 425 down-regulated genes. Biological process annotation and pathway enrichment analysis identified several DCIS-related signaling pathways. Finally, PPI network was constructed with String website in order to get the hub codes involved in Ductal carcinoma in situ. We thus concluded that Five genes: *CDK1*, *CCNB2*, *MAD2L1*, *PPARG*, *ACACB* were finally identified to participate in the regulation and serve as potential diagnosis signatures in in Ductal carcinoma in situ. Finally, complmentarity between *CDK1* and three drugs, Aminophenazone, Pomalidomide and the Rosoxacin, implies novel pharmacological value of those drugs in breast cancer.

## INTRODUCTION

Ductal carcinoma in situ (DCIS) comprises a heterogeneous group of neoplastic lesions confined to the breast ducts [1, 2], which subject to clonal proliferation of epithelial malignant cells yet does not exhibit stromal invasion into adjacent breast stroma under microscopic examination. The increased prevelance of mammographic screening has tremendously changed the situation where DCIS had been underdiagnosed, and the past two decades witnessed the dramatic increase of detection rate. The 10-year cancer-specific survival of DCIS reached over 95%, indicating that early diagnosis would exert substantial influence on prognosis [3]. However, the heterogeneity of DCIS warrants comprehensive investigations into the molecular mechanisms to enlighten management of this disease [4].

Traditionally, the diagnosis of DCIS relied on mammography and clinic pathologic findings [5]. However, the accuracy suffered from high false positive rate and high false negative rate, resulting in under- or over-treatment of DCIS [6]. Therefore, characterization of molecular signatures holds the promise for improving diagnosis of DCIS. Multiple lines of evidence demonstrated that diagnosis, prognosis and therapy prediction gain remarkable improvement from molecular signature [7, 8, 9], particularlyin identifying risk factors and early phase of carcinogenesis. Another compelling advantage of molecular signature resides in its non-invasive operation and relatively low cost, rendering it a promising method for seeking predictive and therapeutic biomarkers.

Microarray has been employed in characterizing themolecular mechanism of DCIS [10, 11, 12]. Several expression profiling researches of DCIS have been released, most of which were designed to identify key candidate genes implicated in the progression of DCIS to invasive ductal breast cancer (IDC) [13]. In this research, we collected 14 microarray data from microarray dataset: normal ductal cells from 5 patients; 9 surgical specimens with DCIS and proceeded several of bioinformatics analysis to recognize the molecular mechanism of DCIS. Gene with expression different between normal cells and cancer cells were identified. *MAD2L1*, *CDK1* and *ACACB* exhibit significantly distinctive expression patterns and may be highly involved in cacer related pathways of breast cancer, DCIS. Furthermore, docking analysis revealed that CDK1, a potential target of DCIS, has active site complementary with three antibiotic drugs, Pomalidomide and the Rosoxacin, indicating novel pharmacological utility of these drugs.

## RESULTS

### Analysis of DEGs

**Figure 1:**
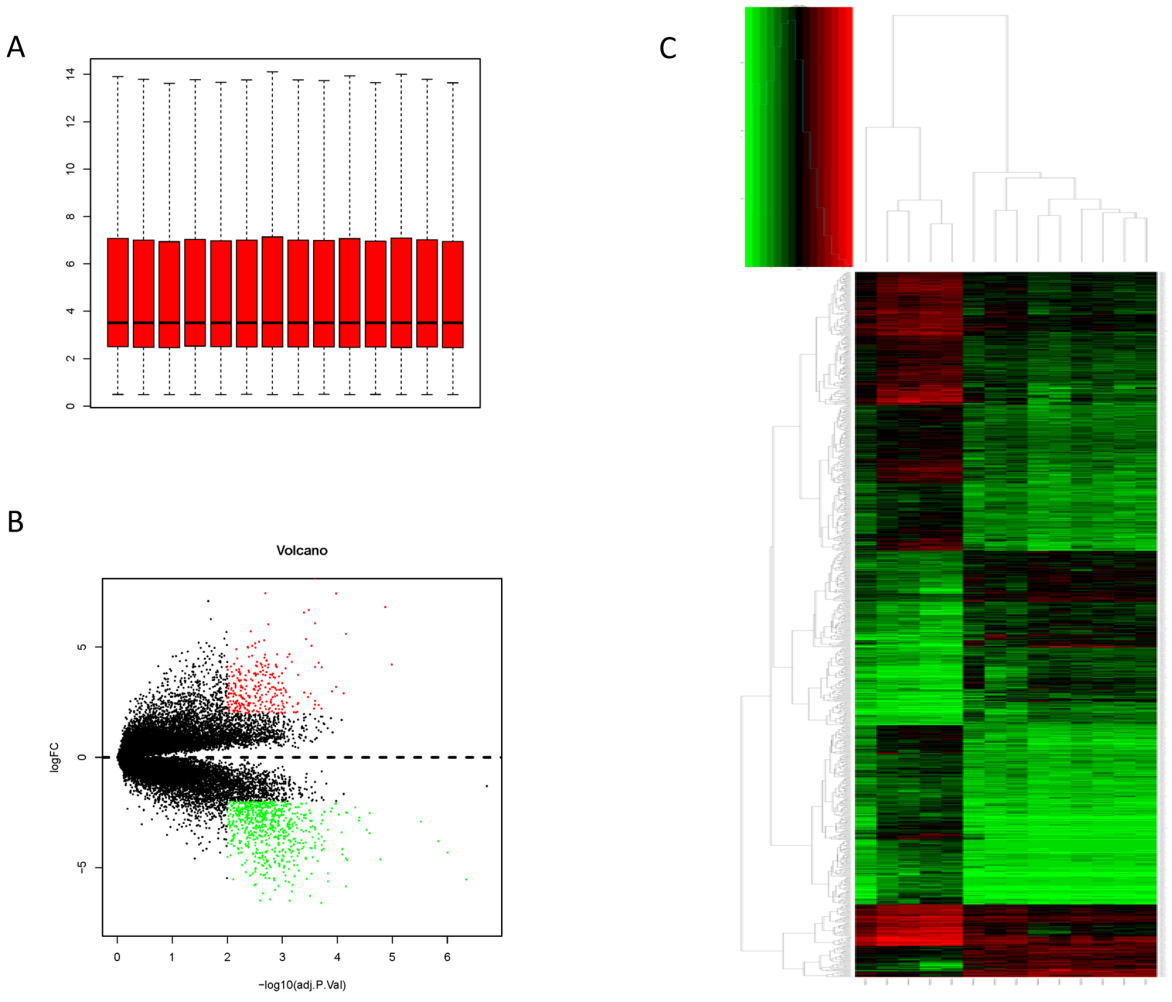
Data process **(A)** data distribution after normalization; **(B)** Hierarchical cluster dendrogram of DEGs; **(C)** The DEGs in breast cancer in situ compared with those in normal samples

The expression profile were preprocessed and then analyzed by Affy package in R language. Total genes were screened. Cassette figures after data standardization was shown in Figure 1A. The alignment of black dots on the same line indicates good standardization.

Hierarchy cluster analysis demonstarted that the 9 breast cancer in situ samples and the 5 normal samples showed different distribution (Figure 1B). The result revealed that grouping was reasonable and the data can be directly applied to further analysis. A total of 649 DEGs were identified in normal ductal cells obtain from DCIS patients. There are 224 up-regulated genes and 425 down-regulated genes (Figure 1C). The top ten up-regulated genes (with the highest log-transformed fold change) were *CEACAM6*, *S100P*, *RRM2*, *COL10A1*, *KMO*, *TFAP2B*, *SDC1*, *COMP*, *KIAA0101* and *GJB2*. The top ten down-regulated genes (with the smallest log-transformed fold change) were *CIDEC*, *PCOLCE2*, *HSPB7*, *ACVR1C*, *PLIN4*, *TUSC5*, *GPD1*, *TIMP4*, *LEP* and *CIDEA* (Table 1).

**Table 1:** The top 10 most up and downregulated genes

Upregulated			Downregulated		
geneNames	logFC	adj	geneNames	logFC	adj
CEACAM6	7.76695	0.000179	CIDEA	-6.50126	0.000733
S100P	7.438669	0.002028	LEP	-6.49356	0.00252
RRM2	6.625876	0.000365	TIMP4	-6.43726	0.001675
COL10A1	6.207364	4.15E-05	GPD1	-6.30837	0.000772
KMO	6.034898	0.001794	TUSC5	-6.28374	0.000312
TFAP2B	5.35991	0.003991	PLIN4	-6.16579	0.002251
SDC1	5.207596	0.002872	ACVR1C	-5.96447	0.000369
COMP	5.057335	0.000294	HSPB7	-5.8322	0.001175
KIAA0101	4.972437	0.003068	PCOLCE2	-5.80062	0.003171
GJB2	4.972225	0.004187	CIDEC	-5.78743	0.000647

### Function and pathway enrichment analysis

**Figure 2:**
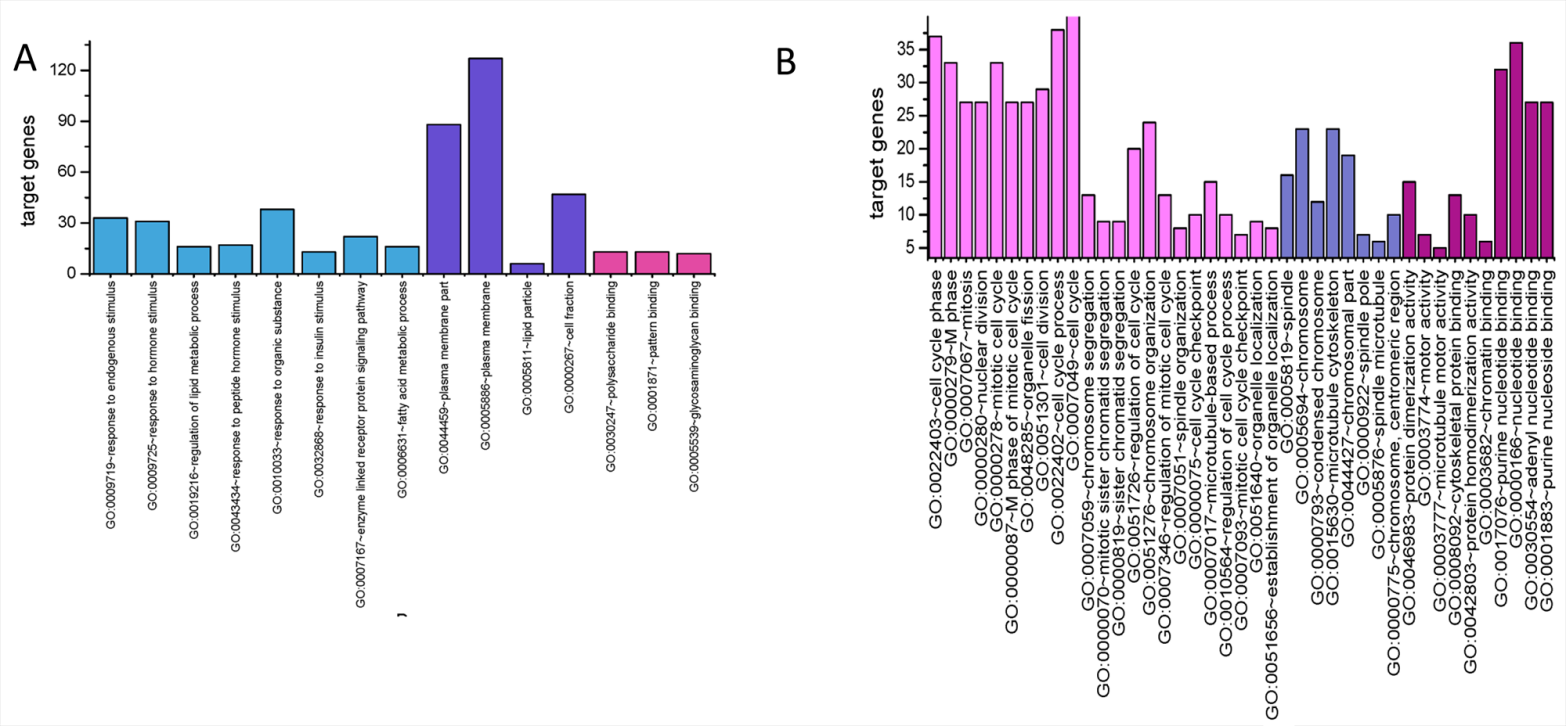
Most significantly enriched GO terms of DEGs according P value

A total of 224 up-regulated genes and 425 down-regulated genes were uploaded to DAVID for GO enrichment (p < = 0.05 as significant). Figure 2A and 2B showed the top enriched GO terms of up- and down-regulated genes separately. The up-regulated genes were mainly enriched in cell cycle phase, M phase, mitosis, nuclear division, mitotic cell cycle, M phase of mitotic cell cycle, organelle fission, cell division, cell cycle process and cell cycle (Table 2), whereas the down-regulated genes were over-represented in response to endogenous stimulus, response to hormone stimulus, regulation of lipid metabolic process, plasma membrane part, plasma membrane, response to peptide hormone stimulus, response to organic substance, response to insulin stimulus, lipid particle and cell fraction, etc. (Table 3). The KEGG pathways of up- and downregulated genes were summarized in Tables 4 and 5. The upregulated genes were mainly enriched in Cell cycle, Oocyte meiosis, Progesterone-mediated oocyte maturation, p53 signaling pathway and Systemic lupus erythematosus (Table 4). Down-regulated genes were related to Glycerolipid metabolism, PPAR signaling pathway, Fatty acid metabolism, Pyruvate metabolism, Insulin signaling pathway, Glycolysis / Gluconeogenesis, Adipocytokine signaling pathway, Histidine metabolism, Propanoate metabolism and Retinol metabolism (Table 5).

**Table 2:** The enriched GO terms of the up-regulation DEGs

Term	Count	P Value	Genes
GO:0022403∼cell cycle phase	37	3.26E-20	PRC1, BLM, NEK2, AURKA, ANLN, PTTG1, FAM83D, CCNA2, CDCA5, HELLS, ASPM, CDCA3, CDC7, CDK1, KIF11, MKI67, SGOL2, DLGAP5, TPX2, CENPF, NUSAP1, BIRC5, CENPE, NDC80, PBK, UBE2C, CDKN3, SMC4, CCNB1, INHBA, MAD2L1, CCNB2, FANCD2, ZWINT, CKS2, BUB1B, KPNA2
GO:0000279∼M phase	33	1.85E-19	PRC1, NEK2, AURKA, ANLN, PTTG1, FAM83D, CCNA2, CDCA5, HELLS, ASPM, CDCA3, CDK1, KIF11, MKI67, SGOL2, DLGAP5, TPX2, CENPF, NUSAP1, BIRC5, CENPE, NDC80, PBK, UBE2C, SMC4, CCNB1, CCNB2, MAD2L1, FANCD2, ZWINT, CKS2, BUB1B, KPNA2
GO:0007067∼mitosis	27	4.93E-18	NEK2, AURKA, ANLN, PTTG1, FAM83D, CCNA2, CDCA5, HELLS, ASPM, CDCA3, CDK1, KIF11, DLGAP5, TPX2, CENPF, NUSAP1, BIRC5, CENPE, NDC80, PBK, UBE2C, SMC4, CCNB1, CCNB2, MAD2L1, ZWINT, BUB1B
GO:0000280∼nuclear division	27	4.93E-18	NEK2, AURKA, ANLN, PTTG1, FAM83D, CCNA2, CDCA5, HELLS, ASPM, CDCA3, CDK1, KIF11, DLGAP5, TPX2, CENPF, NUSAP1, BIRC5, CENPE, NDC80, PBK, UBE2C, SMC4, CCNB1, CCNB2, MAD2L1, ZWINT, BUB1B
GO:0000278∼mitotic cell cycle	33	6.08E-18	PRC1, BLM, NEK2, AURKA, ANLN, PTTG1, FAM83D, CCNA2, CDCA5, HELLS, ASPM, CDCA3, CDC7, CDK1, KIF11, DLGAP5, TPX2, CENPF, NUSAP1, BIRC5, CENPE, NDC80, PBK, UBE2C, CDKN3, SMC4, CCNB1, INHBA, CCNB2, MAD2L1, ZWINT, BUB1B, KPNA2
GO:0000087∼M phase of mitotic cell cycle	27	7.75E-18	NEK2, AURKA, ANLN, PTTG1, FAM83D, CCNA2, CDCA5, HELLS, ASPM, CDCA3, CDK1, KIF11, DLGAP5, TPX2, CENPF, NUSAP1, BIRC5, CENPE, NDC80, PBK, UBE2C, SMC4, CCNB1, CCNB2, MAD2L1, ZWINT, BUB1B
GO:0048285∼organelle fission	27	1.35E-17	NEK2, AURKA, ANLN, PTTG1, FAM83D, CCNA2, CDCA5, HELLS, ASPM, CDCA3, CDK1, KIF11, DLGAP5, TPX2, CENPF, NUSAP1, BIRC5, CENPE, NDC80, PBK, UBE2C, SMC4, CCNB1, CCNB2, MAD2L1, ZWINT, BUB1B
GO:0051301∼cell division	29	7.91E-17	PRC1, NEK2, ANLN, PTTG1, LLGL2, FAM83D, CCNE2, CCNA2, CDCA5, ASPM, HELLS, CDCA3, CDC7, CDK1, KIF11, SGOL2, CENPF, NUSAP1, BIRC5, CENPE, NDC80, UBE2C, SMC4, CCNB1, CCNB2, MAD2L1, ZWINT, CKS2, BUB1B
GO:0022402∼cell cycle process	38	1.12E-16	PRC1, BLM, NEK2, AURKA, ANLN, PTTG1, FAM83D, CCNA2, CDCA5, HELLS, ASPM, CDCA3, CDC7, CDK1, KIF11, MKI67, SGOL2, DLGAP5, TPX2, CENPF, NUSAP1, BIRC5, CENPE, NDC80, PBK, UBE2C, CDKN3, SMC4, CCNB1, INHBA, MAD2L1, CCNB2, FANCD2, ZWINT, CKS2, BUB1B, KPNA2, BARD1
GO:0007049∼cell cycle	43	7.23E-16	PRC1, BLM, NEK2, ANLN, AURKA, PTTG1, LLGL2, FAM83D, CCNE2, FANCI, CCNA2, CDCA5, HELLS, ASPM, CDCA3, CDC7, CDK1, KIF11, MKI67, SGOL2, DLGAP5, TPX2, CENPF, NUSAP1, BIRC5, CENPE, NDC80, PBK, CDKN3, UBE2C, SMC4, CCNB1, INHBA, UHRF1, MAD2L1, CCNB2, MAPK13, FANCD2, ZWINT, CKS2, BUB1B, KPNA2, BARD1
GO:0007059∼chromosome segregation	13	4.59E-10	NEK2, SGOL2, DLGAP5, NUSAP1, CENPF, NDC80, CENPE, BIRC5, PTTG1, SMC4, MAD2L1, ZWINT, CDCA5

**Table 3:** The enriched GO terms of thedown-regulation DEGs

Term	Count	P Value	Genes
GO:0009719∼response to endogenous stimulus	33	2.55E-10	CAV2, RBP4, CAV1, STAT5A, TACR1, ALDOC, PPARG, PFKFB1, FOXO1, PDE3B, GNG11, TIMP4, GNG12, ACVR1C, PRKAR2B, SORBS1, ANGPT1, GNG2, SIK2, GHR, TXNIP, IRS2, CRYAB, ACADS, CDO1, PCK1, LEP, GNAL, PLA2G4A, ADM, ALDH2, TGFBR3, CA4
GO:0009725∼response to hormone stimulus	31	4.25E-10	CAV2, RBP4, CAV1, STAT5A, TACR1, PPARG, PFKFB1, FOXO1, PDE3B, GNG11, TIMP4, GNG12, ACVR1C, PRKAR2B, SORBS1, ANGPT1, GNG2, SIK2, GHR, TXNIP, IRS2, CRYAB, ACADS, CDO1, PCK1, LEP, PLA2G4A, ADM, ALDH2, TGFBR3, CA4
GO:0019216∼regulation of lipid metabolic process	16	1.89E-08	CAV1, IRS2, THRB, STAT5A, PPARG, MLXIPL, CIDEA, PDE3B, PNPLA2, ACACB, LEP, AGTR1, PLA2G4A, ACSL1, SORBS1, BMP6
GO:0044459∼plasma membrane part	88	3.71E-08	DLC1, CYB5R3, GYPC, TLN2, TSPAN4, TACR1, FERMT2, CLDN5, TSPAN7, CPEB1, KCNIP2, ITSN1, TENC1, DDR2, AMOTL2, CALB2, ACVR1C, LNPEP, EDNRB, AGTR1, GPC3, SDPR, GNG2, SLC4A4, GPIHBP1, SAMD4A, GHR, TYRO3, PTGER3, F10, LIFR, SLC7A10, SSPN, NCAM1, TNS1, CD36, EGFLAM, PGM5, PTRF, SGCG, CD34, CD99L2, CA4, JAM2, STBD1, FXYD1, CAV2, FGFR1, PALM, CAV1, EMCN, GNAI1, ENPP2, ADCYAP1R1, MRAS, MMD, MME, GNG11, GNG12, ALDH3A2, SLC29A1, SORBS1, PPL, DMD, SYN2, ADRA2A, PRIMA1, RASA3, EHD2, PTPRB, KL, MAOA, KCNB1, ITGA1, ANXA1, NPR1, ATP1A2, LYVE1, TMEM47, SLC16A7, ITGA7, NTRK2, SPTBN1, SCN4B, TGFBR3, PDZD2, SCARA5, LIPE
GO:0005886∼plasma membrane	127	2.11E-07	DLC1, PLXNA4, GLDN, TSPAN4, TSPAN7, ITSN1, AMOTL2, CALB2, AGTR1, ELTD1, GNG2, SLC4A4, SAMD4A, TYRO3, IRS2, F10, CRYAB, LIFR, PNPLA2, SSPN, NCAM1, TNS1, CD36, EGFLAM, PGM5, KCNT2, PTRF, CD34, CD300LG, EMP1, ABCA8, FXYD1, CAV2, GPR146, FGFR1, PALM, EMCN, CAV1, MRAP, GNAI1, ENPP2, ADCYAP1R1, MRAS, MMD, AKAP12, MME, NRN1, SLC29A1, CDC42EP2, PPL, DMD, ADRA2A, PRIMA1, STX11, LPL, KLB, MAOA, KCNB1, ITGA1, MCAM, PCDH19, PCDH18, LYVE1, ITGA7, NTRK2, SPTBN1, TGFBR3, PDZD2, SCARA5, CYB5R3, GYPC, TLN2, TACR1, FERMT2, CLDN5, CPEB1, KCNIP2, TENC1, DDR2, ACVR1C, LNPEP, EDNRB, SPRY2, WISP2, GPC3, SDPR, QKI, CAT, GPIHBP1, GHR, PTGER3, STXBP1, SLC7A10, PCDH9, GNAL, SGCG, CD99L2, CA4, PCYOX1, JAM2, STBD1, CHL1, AOC3, GNG11, GNG12, ZBTB16, ALDH3A2, RGMA, ACSL1, SORBS1, PLIN4, SYN2, RASA3, PPAP2B, EHD2, PTPRB, PLA2G16, KL, ANXA1, NPR1, ATP1A2, P2RY12, TMEM47, SLC16A7, SCN4B, LIPE, GPR116
GO:0043434∼response to peptide hormone stimulus	17	2.37E-07	RBP4, CAV2, IRS2, STAT5A, PPARG, PFKFB1, FOXO1, PDE3B, TIMP4, CDO1, PCK1, ACVR1C, LEP, ADM, SORBS1, SIK2, GHR
GO:0010033∼response to organic substance	38	9.90E-07	CAV2, RBP4, CAV1, STAT5A, TACR1, ALDOC, PFKFB1, PPARG, PDE3B, FOXO1, GNG11, TIMP4, GNG12, ACVR1C, PRKAR2B, ACSL1, SORBS1, GSN, GNG2, ANGPT1, SIK2, GHR, TXNIP, IRS2, CRYAB, ACADS, LIFR, CDO1, PCK1, LEP, GNAL, PLA2G4A, ADM, HSPB7, HSPB2, ALDH2, TGFBR3, CA4
GO:0032868∼response to insulin stimulus	13	1.69E-06	RBP4, IRS2, PFKFB1, PPARG, FOXO1, PDE3B, PCK1, ACVR1C, LEP, ADM, SORBS1, SIK2, GHR
GO:0005811∼lipid particle	6	1.42E-05	CAV2, CAV1, PLIN1, PLIN4, CIDEA, PNPLA2
GO:0007167∼enzyme linked receptor protein signaling pathway	22	1.89E-05	TXNIP, FGFR1, BMP2, IRS2, NDN, KL, STAT5A, ADCYAP1R1, LIFR, FOXO1, DDR2, CHRDL1, SORBS1, NTRK2, GDF10, SPTBN1, TGFBR3, ANGPT1, FGF2, SIK2, BMP6, GHR
GO:0000267∼cell fraction	47	2.14E-05	CAV2, FGFR1, CAV1, MMD, MME, PDE3B, NMB, ITSN1, LNPEP, SLC29A1, PRKAR2B, EDNRB, WISP2, ACSL1, FMO2, DMD, CYP26B1, GPX3, LMOD1, RAPGEF3, EHD2, SAMD4A, GPD1, F10, CRYAB, KL, ITGA1, FADS3, GYG2, ATP1A2, SOD3, PCK1, PLA2G4A, LYVE1, CD36, PTRF, DGAT2, ADM, SLC16A7, PYGL, FBLN5, CLIC5, HSPB2, CA4, STBD1, EMP1, PC

**Table 4:** The enriched pathways of the up-regulation DEGs

Term	Count	P Value	Genes
hsa04110:Cell cycle	9	1.49E-04	CDC7, CCNE2, CCNB1, CDK1, MAD2L1, CCNB2, BUB1B, PTTG1, CCNA2
hsa04114:Oocyte meiosis	7	0.002418	CCNE2, CCNB1, CDK1, MAD2L1, CCNB2, AURKA, PTTG1
hsa04914:Progesterone-mediated oocyte maturation	6	0.004289	CCNB1, CDK1, MAD2L1, CCNB2, MAPK13, CCNA2
hsa04115:p53 signaling pathway	5	0.01018	CCNE2, CCNB1, CDK1, CCNB2, RRM2
hsa05322:Systemic lupus erythematosus	5	0.03529	HIST1H2BD, HIST1H2BE, HIST2H2BE, HIST1H2BH, HIST1H4J

**Table 5:** The enriched pathways of the down-regulation DEGs

Term	Count	P Value	Genes
hsa00561:Glycerolipid metabolism	8	1.29E-04	LPL, DGAT2, ALDH2, MGLL, GPAM, PPAP2B, ALDH3A2, AGPAT2
hsa03320:PPAR signaling pathway	9	3.49E-04	LPL, CD36, ACSL1, SORBS1, PLIN1, PPARG, ACADL, PCK1, ANGPTL4
hsa00071:Fatty acid metabolism	6	0.003354	ACSL1, ACADS, ADH1C, ALDH2, ADH1B, ACADL, ALDH3A2
hsa00620:Pyruvate metabolism	6	0.003354	ALDH2, ACACB, ACSS2, ALDH3A2, PC, PCK1
hsa04910:Insulin signaling pathway	10	0.007669	PRKAR2B, IRS2, INPP5K, SORBS1, PYGL, PDE3B, FOXO1, ACACB, LIPE, PCK1
hsa00010:Glycolysis / Gluconeogenesis	6	0.018507	ALDOC, ADH1C, ALDH2, ADH1B, ACSS2, ALDH3A2, PCK1
hsa04920:Adipocytokine signaling pathway	6	0.028403	LEP, IRS2, CD36, ACSL1, ACACB, PCK1
hsa00340:Histidine metabolism	4	0.037481	ASPA, MAOA, ALDH2, ALDH3A2
hsa00640:Propanoate metabolism	4	0.04814	ALDH2, ACACB, ACSS2, ALDH3A2
hsa00830:Retinol metabolism	5	0.049434	DHRS3, DGAT2, CYP26B1, ADH1C, ADH1B, RDH5

### Analysis of PPI network

**Figure 3:**
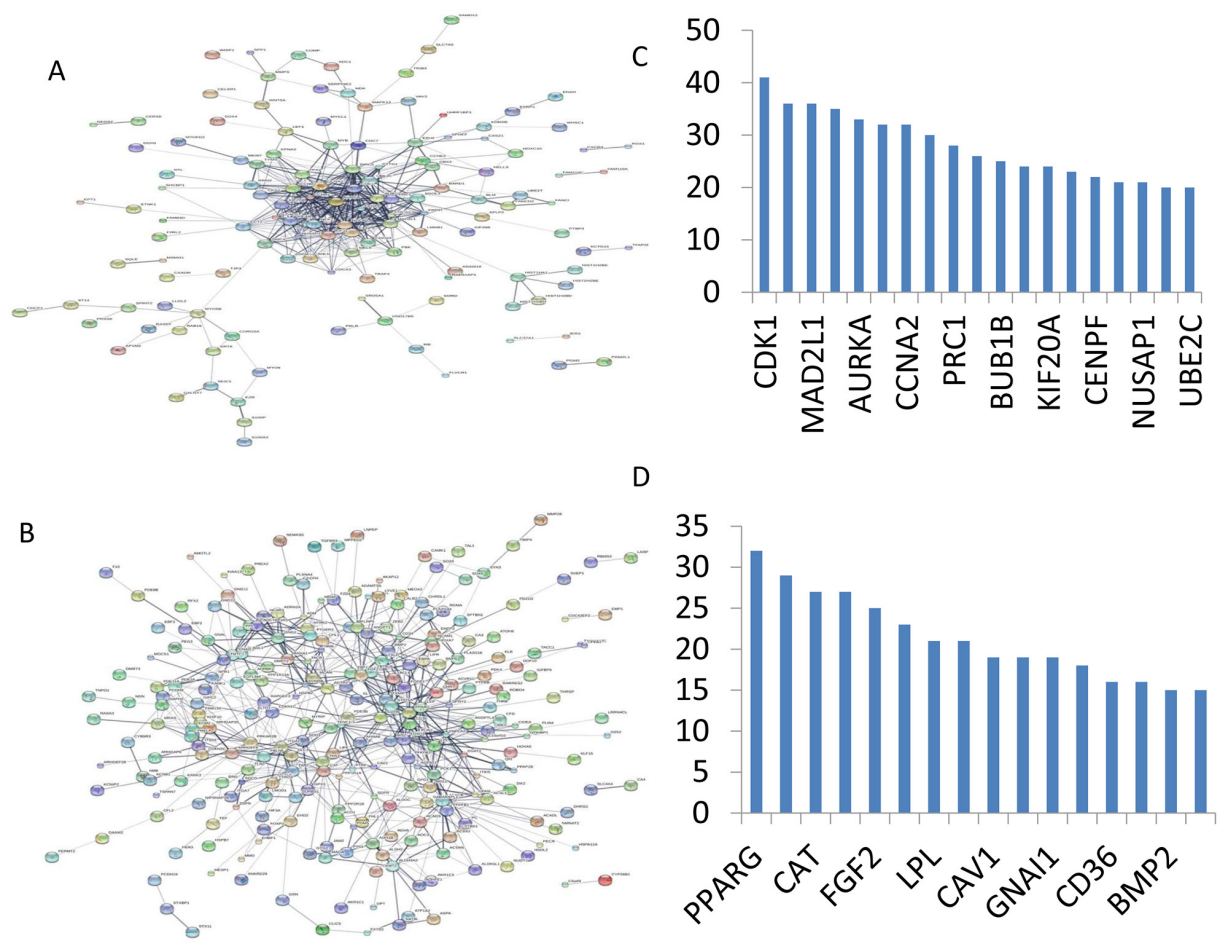
PPI network of DEGs obtained from the STRING database **(A)** PPI network of DEGs of upregulation DEGs; **(B)** PPI network of DEGs of downregulation DEGs; **(C)** Hub nodes in the PPI network constructed with upregulated genes; **(D)** Hub nodes in the PPI network constructed with downregulated genes.

Initially, to get PPI data, we uploaded 478 DEGs to STRING website. Next, the samples whose PPI score above 0.4 were selected to construct PPI networks. The PPI networks of up- and down-regulated DEGs were displayed in Figure 3. The up-regulated network was created with 182 nodes and 486 edges (Figure 3A). The proteins cyclin dependent kinase 1 (CDK1, degree = 41), cyclin B2 (CCNB2, degree = 36), and MAD2 mitotic arrest deficient-like 1 (MAD2L1, degree = 36) were hub nodes in this network. The down-regulated PPI network was constructed with 262 nodes and 633 edges (Figure 3B). The protein superoxisome proliferator activated receptor gamma (PPARG, degree = 32), acetyl-CoA carboxylase beta (ACACB, degree = 29) and catalase (CAT, degree = 27) were hub nodes in this network (Figure 3C and 3D). These genes are also enriched in Go terms and KEGG pathway excluding *CAT* gene.

### Key genes filter and survival analysis

**Figure 4:**
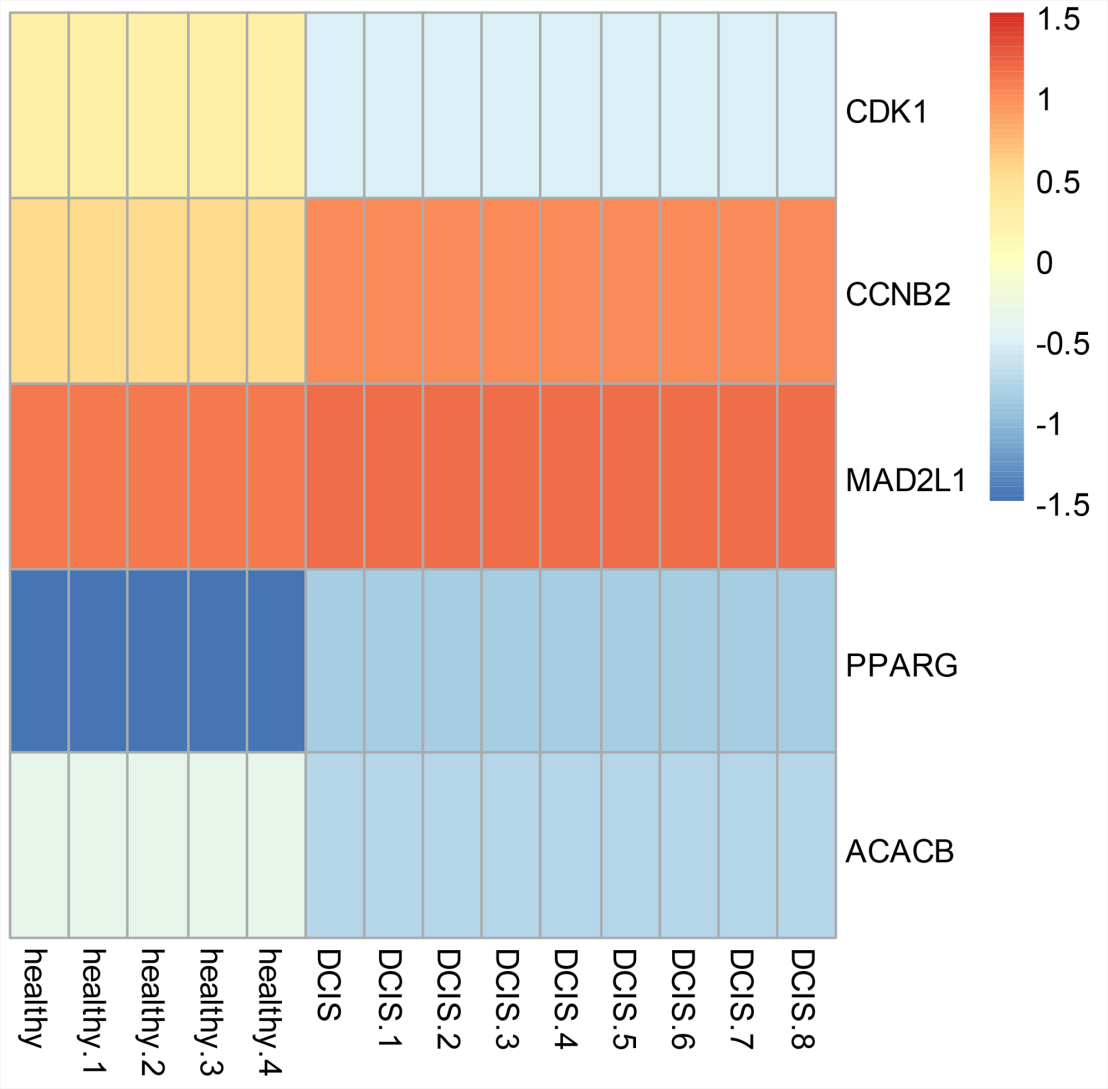
Heatmap of hub genes in PPI network and the corresponding enriched GO and KEGG terms

To visualize gene expression level of the 5 most intersecting genes: *CDK1*, *CCNB2*, *MAD2L1*, *PPARG*, and *ACACB*, we used pheatmap package implemented in R to generate a heatmap (Figure 4) to detect the gene expression differences between normal ductal cells and DCIS. In this step, we targeted three genes: *MAD2L1*, *CDK1* and *ACACB*, because they showed the most significant distinction in gene expression profile.

### Docking the ZINC database

**Figure 5:**
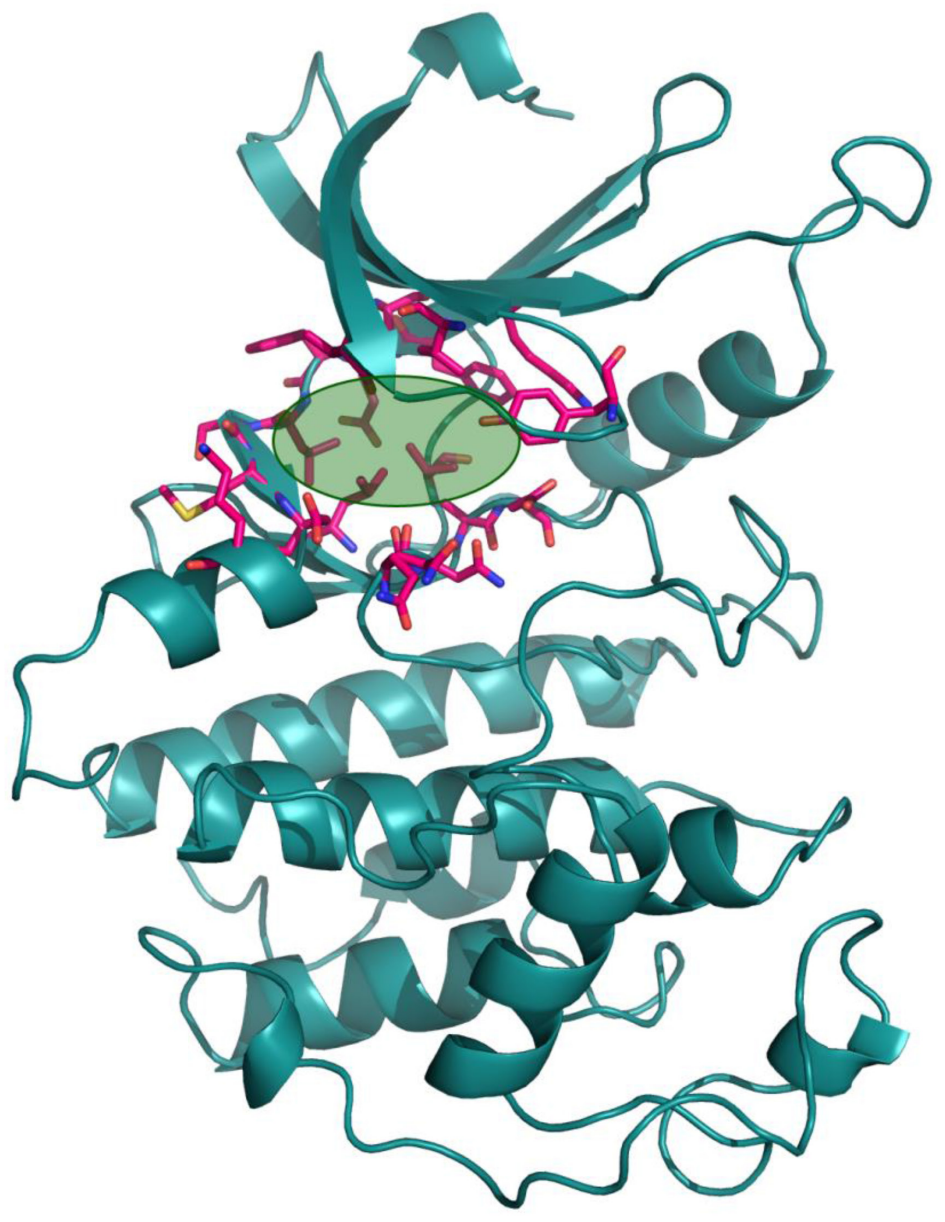
The structure of the CDK1 The active site of CDK1 identified by the FTMAP server was depicted by lime circle.

**Figure 6:**
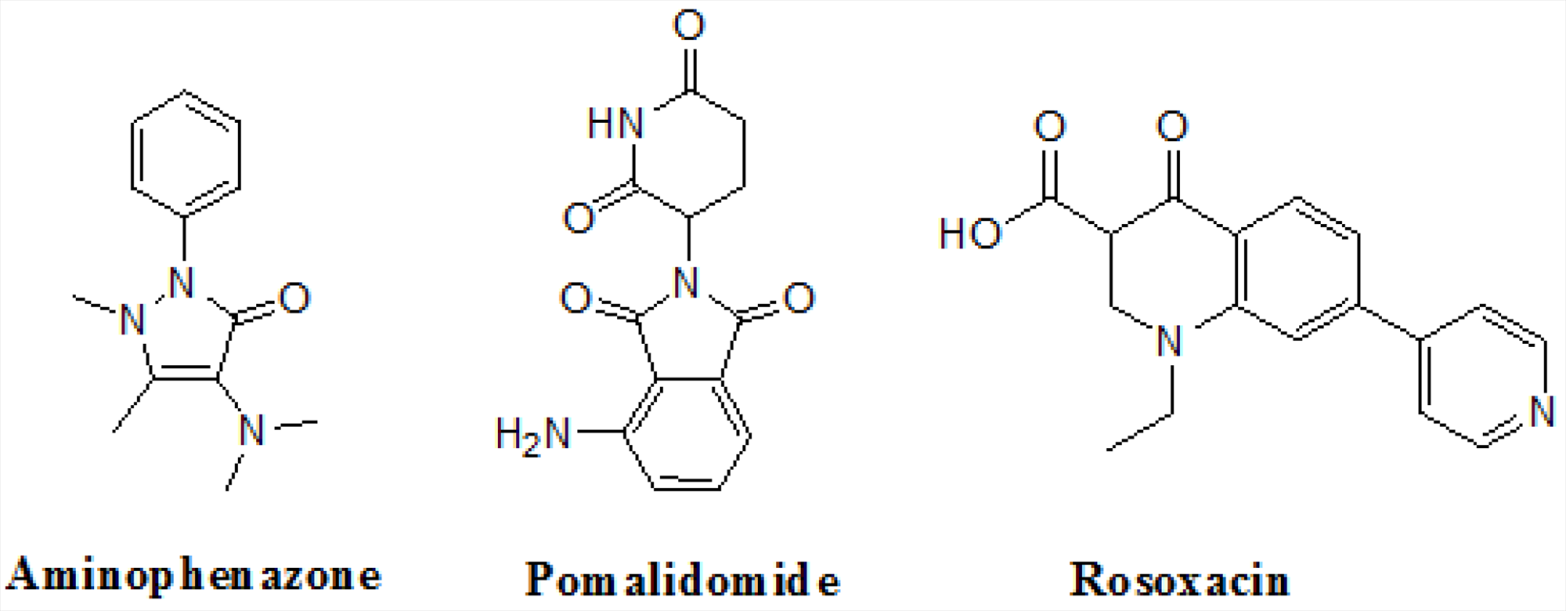
The structures of the Aminophenazone, Pomalidomide and Rosoxacin The Aminophenazone was a pyrazolone with analgesic, anti-inflammatory, and antipyretic properties but has risk of agranulocytosis. Pomalidomide, an analogue of thalidomide, is an immunomodulatory antineoplastic agent. FDA approved on February 8, 2013. Rosoxacin is a quinolone derivative antibiotic for the treatment of bacterial infection of respiratory tract, urinary tract, GI, CNS and immuno compromised patients.

The 26,504 molecules of the lead-like subset of the ZINC database were docked into the structure of CDK1. The molecules were scored for receptor complementarity based on the sum of their van der Waals [using the AMBER potential] and electrostatic interaction energies [using ligand probe charges in an electrostatic potential calculated by DelPhi, corrected for ligand desolvation (Figure 5). Based on their docking scores, the compounds were ranked from best to worst fitting, and all of the compounds prioritized for experimental testing were selected from the top-ranking 500 molecules, representing 0.05% of the docked library. In addition, these molecules were inspected visually for features not captured in the docking calculation, such as chemical diversity, actual commercial availability, and an overall balance between polar and nonpolar complementarily to the binding site. Finally, 9 molecules were selected for the further study (Table 6). Moreover, a structure alignment between the selected 9 compounds and the members of the DrugPort containing 1492 approved drugs were performed to find FDA approved drugs which can inhibit the activity of the CDK1. Consequently, Aminophenazone, Pomalidomide and the Rosoxacin shared large similarity to the structures of the Zinc210393, Zinc 312408 and Zinc5316172, respectively, suggesting that these compounds can bind to the active site of the CDK1 with the similar poses of the Zinc210393, Zinc 312408 and Zinc5316172 (Figure 6). It was postulated that Aminophenazone, Pomalidomide and the Rosoxacin might have therapeutic value in treating diseases derived from the dysfunction of CDK1.

**Table 6:** Top-10 docking result

ZINC ID	Chemicals	Score (kcal/mol)
Zinc320022	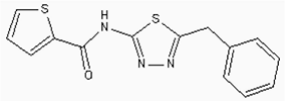	-15.2
Zinc19796871	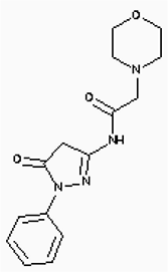	-16.1
Zinc210393	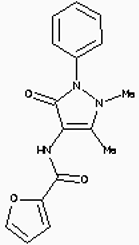	-14.2
Zinc368131	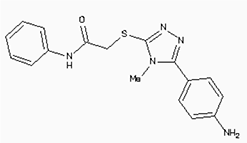	-15.6
Zinc123806	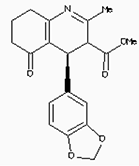	-16.7
Zinc372776	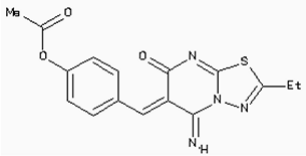	-15.2
Zinc291147	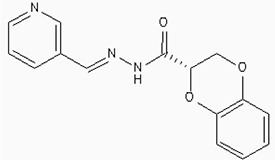	-13.9
Zinc5316172	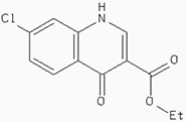	-14.7
Zinc312408	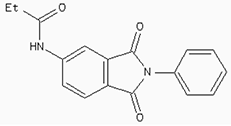	-15.6
Zinc42568	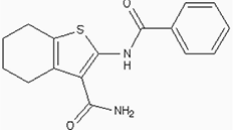	-14.5

## DISCUSSION

In our research, the gene expression profile information of GSE21422 was downloaded from GEO database to determine DEGs between DCIS and healthy breast tissues utilizing bioinformatics tools. Entirely, 649 DEGs including 224 up- and 425 down-regulated genes were selected. The functional enrichment analysis results revealed that up regulated genes were involved in cell cycle and Oocyte development, p53 signaling pathway, while down-regulated genes were mainly enriched in the lipid metabolism and PPAR pathway.

Moreover, Five genes were hub nodes in PPI networks. Expression profile showed that *MAD2L1*, *CDK1* and *ACACB*exhibit most distinctive expression pattern. These DEGs and their related functions may be involved in DCIS progression. MAD2L1, also termedas Mitotic Arrest Deficient 2-like 1, is an essential component in mitosis when chromosomes are detached to the mitotic spindle that maintains openning of chromosomes, and is involved in the spindle checkpoint during mitosis [17, 18, 19]. It has great potential in assessment of prognosis and may serve as tumor biomarkers in breast cancer [20]. In this research, we specifically identified a subset of DCIS in which MAD2L1 plays as a biomarker. CDK1 is a member of the Ser/Thr protein kinase family [21, 22]. In this study, over-expression of CDK1 was enriched in Oocyte development and p53 signaling pathwayindicating that overexpression of CDK1 may promote DCIS progression by p53 signaling. Additionally, ACACB is a complex multifunctional enzyme system which determines the catalytic rate of fatty acid oxidation [23, 24, 25]. However, current studies characterizing the function of ACACB in DCIS is rare. The current research revealed that the ectopic expression of ACACB reminiscent of its clinical value as a diagnostic indicator of DCIS [26, 27, 28]. We also demonstrated that the active site of CDK1 is complementary with three FDA approved drugs Aminophenazone, Pomalidomide and the Rosoxacin, indicating novel pharmacological value beyond their antibiotic function. Although more thorough researches are warranted, anti-cancer therapeutical potency of these three drugs in breast cancer may remain to be discovered.

In conclusion, our research identified a general amount of 478 DEGs which may be associated with pathogenesis and progression of DCIS. Functional pathway enrichment analysis and PPI network construction were combined to identify three genes with significantly distinctive expression pattern and highly connecting with cancer related pathways. These genes could be a critical part in the progression of IDC and serve as prognosis indicators. Complmentarity between CDK1 and three drugs, Aminophenazone, Pomalidomide and the Rosoxacin, implies novel pharmacological value of those drugs in breast cancer.

## METHODS

### Microarray data

The microarray data GSE21422 was collected from Gene Expression Omnibus(GEO) which was built upon GPL570 platform. This platform was stored by Schaefer C et al [13] and houses 19 microarray data of nine DCIS, 5 invasive ductal carcinoma(IDC) and 5 healthy control samples obtained from patients with breast reduction surgery.

### Data preprocessing

The original CEL data were imported into R and affy package was implemented for background correction and normalization. The expression of genes corresponding to multi probes were summarized. mas5calls in Affy was run to filter out samples with no gene expression.

### Differentially expressed genes selection

DEGs between 5 healthy samples and 9 DCIS samples were identified using Limma package [14]. The FDR was set to 0.01 and those genes with |log_2_ fold change | > =2 were regarded as differentially expression genes (DEGs).

### Functional annotation and pathway analysis of DEGs

Database for Annotation, Visualization, and Integrated Discovery (DAVID) is a web-server that combines functional genomic annotations with intuitive graphical summaries [15]. Gene lists or protein identifiers were rapidly annotated and categorized using comprehensive categorical data from Gene Ontology (GO), protein domain, and biochemical pathway membership. To investigate the inter-connection between pathways involved in pathogical mechanism of DCIS, GO and Pathway enrichment analysis on DEGs were performed with the DAVID analysis system, significance level p<= 0.05.

### Protein interaction networks analysis

Search Tool for the Retrieval of Interacting Genes/Proteins (STRING) [16] database (http://string-db.org/) was used to analyze protein interactions. STRING has advantages in that it aggregates most of the available information on protein-protein associations, which were benchmarked and scored against a common reference of functional partnership annotated at KEGG. There are more than 1100 organisms in extensive protein connection with global data. In this research, protein-protein interaction (PPI) network of DEGs was constructed based on STRING database where the interaction score above 0.4 was considered as *de facto* interaction.

### Protein preparation for docking

The structure of the CDK1 in complex with CYCLINB1 and CKS2 [PDB ID: 4YC3] was used in the docking calculations. The cofactors, ions and water molecules were removed from this complex. then the CYCLINB1 and CSK2 were also removed. The hydrogen atoms of the protein were added and optimized by the REDUCE. The active site of the CDK1 was obtained from the FTMAP server, the orientations for hydroxy groups in selected binding residues were modified to conform to the proton positions determined by the HBUILD module in CHARMM.

### Docking calculations

Virtural screening was performed to identify molecules that could bind to the active site of the CDK1. Docking of all compounds (26,504 compounds) downloaded from the lead-like subset of ZINC database was performed using DOCK 3.6 program. Complementarity of each ligand pose is scored as the sum of the receptor-ligand electrostatic and van der Waals interaction energy and corrected for ligand desolvation. Partial charges from the united-atom AMBER force field were used for all receptor atoms except for Serine, for which the dipole moment was increased as previously described to boost electrostatic scores for poses in polar contact with these important residues.
